# Metabolomic changes in polyunsaturated fatty acids and eicosanoids as diagnostic biomarkers in *Mycobacterium avium* ssp. *paratuberculosis* (MAP)-inoculated Holstein–Friesian heifers

**DOI:** 10.1186/s13567-022-01087-0

**Published:** 2022-09-02

**Authors:** Emma N. Taylor, Manfred Beckmann, Glyn Hewinson, David Rooke, Luis A. J. Mur, Ad P. Koets

**Affiliations:** 1 Institute of Biological, Environmental and Rural Sciences (IBERS), Aberystwyth University, Ceredigion, SY23 3DA UK; 2 Centre of Excellence for Bovine Tuberculosis, Aberystwyth University, Ceredigion, SY23 3DA UK; 3 ProTEM Services Ltd, Horsham, RH12 4BD West Sussex UK; 4 Wageningen Bioveterinary Research, 8221 RA Lelystad, The Netherlands; 5 Faculty of Veterinary Medicine, Population Health Systems, Utrecht University, 3584 CS Utrecht, The Netherlands

**Keywords:** *Mycobacterium avium* subsp. *paratuberculosis*, metabolomics, alpha-linoleic acid and linoleic acid metabolism, polyunsaturated fatty acids, eicosanoids

## Abstract

*Mycobacterium avium* subspecies *paratuberculosis* (MAP) is the causative organism of Johne’s disease, a chronic granulomatous enteritis of ruminants. We have previously used naturally MAP-infected heifer calves to document metabolomic changes occurring in MAP infections. Herein, we used experimentally MAP-inoculated heifer calves to identify biomarkers for MAP infections. At 2-weeks of age, 20 Holstein–Friesian (HF) calves were experimentally inoculated with MAP. These calves, along with 20 control calves, were sampled biweekly up to 13-months of age and then monthly up to 19-months of age. Sera were assessed using flow infusion electrospray high-resolution mass spectrometry (FIE-HRMS) on a Q Exactive hybrid quadrupole-Orbitrap mass spectrometer for high throughput, sensitive, non-targeted metabolite fingerprinting. Partial least squares-discriminate analysis (PLS-DA) and hierarchical cluster analysis (HCA) discriminated between MAP-inoculated and control heifer calves. Out of 34 identified metabolites, six fatty acyls were able to differentiate between experimental groups throughout the study, including 8, 11, 14-eicosatrienoic acid and cis-8, 11, 14, 17-eicosatetraenoic acid which were also detected in our previous study and so further suggested their value as biomarkers for MAP infection. Pathway analysis highlighted the role of the alpha-linoleic acid and linoleic acid metabolism. Within these pathways, two broad types of response, with a rapid increase in some saturated fatty acids and some n-3 polyunsaturated fatty acids (PUFAs) and later n-6 PUFAs, became predominant. This could indicate an initial anti-inflammatory colonisation phase, followed by an inflammatory phase. This study demonstrates the validity of the metabolomic approach in studying MAP infections. Nevertheless, further work is required to define further key events, particularly at a cell-specific level.

**Supplementary Information:**

The online version contains supplementary material available at 10.1186/s13567-022-01087-0.

## Introduction

Johne’s disease, caused by *Mycobacterium avium* subspecies *paratuberculosis* (MAP), is a chronic granulomatous enteritis of ruminants. MAP is frequently transmitted via the ingestion of colostrum, milk and faeces [1]. Following exposure to MAP, infected cattle enter a prolonged incubation period prior to the onset of shedding and clinical signs, such as; weight loss, diarrhoea and reduced milk yields [2]. With estimated herd prevalence’s exceeding 50% in countries with intensive dairy production systems [3] and 10-year annual losses of ~$49 million (~£35 million) in the UK [4], MAP represents an on-going economic and animal welfare challenge for farmers.

Identifying MAP-infected young stock is particularly challenging due to an absence of clinical signs [5], and detection being based on bacterial shedding patterns that are influenced by the level and age of calves at MAP exposure [6]. Individual faecal PCR tests and serum ELISAs are commonly used to identify MAP-infected cattle [7] but exhibit poor sensitivity values of approximately 4% and 13% in light-to-moderate faecal shedders [8]. Furthermore, interferon-γ tests only detect approximately half of MAP faecal culture positive cattle aged between 12 and 23 months of age [9] and milk ELISA tests are limited to post-partum use only. Consequently, a diagnostic tool capable of detecting MAP-infected youngstock would benefit farmers worldwide.

Omic approaches encapsulate various subdivisions of systems biology, including genomics, proteomics, lipidomics and metabolomics [10]. The application of these approaches in MAP research has highlighted multiple potential biomarkers, but these findings require further validation [11–13]. Proteomic approaches identified antigenically distinct recombinant MAP cell envelope proteins; Sdh A and MAP1233m and their detection using ELISA showed maximum sensitivity and specificity values of 94% and 100%, respectively [12]. Further lipidomic approaches were used to show that glycopeptidolipids are replaced by lipopentapeptides within the MAP cell wall. Subsequently, a lipopentapeptide L5P-based test exhibited a sensitivity of 82% [11]. These studies suggest that the performance of these omic study-derived biomarkers exceeds many commercially available tests [8]. This stated, these have focused on post-partum cattle [12, 13] so that the application of these biomarkers in youngstock is questionable.

In contrast, metabolomic studies have focused on both calves and young stock. Analysis of sera from experimentally MAP-infected calves using H^1^ nuclear magnetic resonance (NMR) spectrometry showed metabolomic changes indicative of energy shortages, elevated lipid metabolism and increased protein turnover [14]. Similar energy and lipid-related changes were reported within the sera of mature, naturally MAP-infected cows following analysis via direct analysis in real time coupled with high resolution mass spectrometry (DART-HRMS) [15]. However, the metabolites identified by de Buck et al. [14] exhibited similar levels between MAP-infected and control cattle until 200 days post-infection. Likewise, the metabolites identified by Tata et al. [15] showed overlapping levels between infected, infectious and control groups. Thus, the targeted metabolites were unsuitable as biomarkers for MAP infections.

We have previously examined the sera metabolite profiles of naturally MAP-infected Holstein–Friesian (HF) heifer calves compared to controls between 1 and 19 months of age and identified 33 metabolites which were differentially accumulated in MAP-infected heifer calves [16]. Crucially, five of these metabolites were elevated throughout the study. However, to more clearly examine the time dependent changes following MAP infection in youngstock, we herein use metabolomics to assess a complementary experiment based on HF heifer calves experimentally inoculated with MAP. Biweekly sera samples of heifer calves up to 13 months of age and monthly sera samples of heifer calves up to 19 months of age were analysed using flow infusion electrospray high-resolution mass spectrometry (FIE-HRMS).

## Materials and methods

### Animal samples

The animal experiments were approved by the Animal Welfare Body of Utrecht University (permit number 0202.0806) in accordance with the Dutch regulations on animal experimentation. All samples were bio-archived serum samples previously described in Koets et al. [17].

Sera were obtained from 20 experimentally MAP-inoculated, and 20 controls heifer calves, sampled biweekly until 12 months of age and by monthly until 19 months of age. Control HF heifer calves were sourced at approximately 2 weeks of age from MAP-negative herds. The MAP status of control farms was confirmed via faecal culture from cattle ≥ 24 months of age every 6 months or 12 months for a minimum of 8 years as part of a Dutch national MAP surveillance program. All MAP-inoculated heifer calves were confirmed to be MAP positive in at least one tissue sample at necropsy. No macroscopic lesions were observed.

MAP-inoculated calves were orally inoculated with approximately 1.8 × 10^4^ colony forming units (cfu), supplied across nine doses of 20 g of faeces, and mixed with 100 mL milk replacer over 21 days [17]. The faeces were derived from a MAP-infected cow, whose infection status was confirmed via faecal culture and PCR. Faecal samples were collected from all heifer calves 0-, 14-, 126-, 280-, 406- and 532- days post-inoculation. Of the MAP-inoculated heifer calves, 13 were MAP faecal culture positive and 7 were negative, all control heifer calves were culture negative. MAP faecal culture positive heifer calves displayed faecal shedding at either 14 (one heifer calf) or 126 (twelve heifer calves) days post-inoculation. Faecal samples from heifer calves were processed using the routine diagnostic MAP faecal culture system at the Veterinary Health Service laboratory (Royal GD), Deventer, the Netherlands.

Heifer calves were screened using a commercially available absorbed ELISA assay following the manufacturer’s instructions (Institute Pourquier, Montpellier, France) using blood samples collected 0-, 14-, 126-, 280-, 406- and 532- days post-inoculation. Samples with a sample to negative (S/N) ratio > 59% were considered MAP positive. Of the MAP-inoculated calves, 8 were ELISA positive and 12 were negative. Of the control calves, 2 were ELISA positive and 18 were negative. All positive ELISA results occurred a minimum of 406 days post-inoculation.

### Untargeted metabolite fingerprinting by flow infusion electrospray ionization high resolution mass spectrometry (FIE-HRMS)

Sera were prepared as described by Beckmann et al. [18] with minor amendments. Samples were defrosted on ice, vortexed for 5 s and 200 µL was pipetted into 1520 µL pre-chilled solvent mix (methanol/chloroform [4/1 v/v]) containing 1 micro-spoon of glass beads glass beads (< 106 μm diameter, Sigma, UK). Samples were then vortexed for 5 s, shaken for 15 min at + 4 °C and kept at − 20 °C for 20 min. Following centrifugation at 21 000×*g* and 4 °C for 5 min, 100 µL of the plasma supernatant was transferred into mass spectrometry vials along with 100 µL methanol/water [70/30 v/v]. Samples were stored at -80 °C until analysis using flow infusion electrospray ionization high-resolution mass spectrometry (FIE-HRMS). For each sample, 20 µL were injected into a flow of 60 µL per minute water–methanol, at a ratio of 70% water and 30% methanol, using a Surveyor flow system into a Q Exactive plus mass analyser instrument with UHPLC system (Thermo Fisher Scientific©, Bremen, Germany) for high throughput FIE-HRMS. Data acquisition for each serum sample was done by alternating the positive and negative ionisation modes, throughout four different scan ranges (15–110 m*/z*, 100–220 m*/z*, 210–510 m*/z*, 500–1200 m*/z*) with an acquisition time of 2 min.

### Statistical analysis

Metabolomic data were analysed using MetaboAnalyst 4 [19]. Data were subjected to interquartile range-based filtering, log_10_ transformations and Pareto scaling. Time series analyses used false-discovery rate (FDR) adjusted two-way ANOVA tests to identify *m/z* which significantly (*p*-values < 0.05) differed between experimental classes. Partial least squares-discriminate analysis (PLS-DA) were used to visualise the differences between the experimental classes. Variables of importance for the projection (VIP) scores (> 1) following multivariate analyses were also used to indicate *m/z* which discriminated between the classes. Random forest (RF) was used as an alternative multivariate classification test. The major sources of variation were displayed using unsupervised hierarchical clustering analysis (HCA). Area under the curve (AUC) assessments, based on sensitivity and specificity estimates, were used use to determine the accuracy of the target *m/z* as potential biomarkers.

Significant *m/z* were identified based on accurate mass (5 ppm) using the DIMEdb database [20] based on their ionised masses, molecular formula and the Bovine Metabolome Database [21]. All isotopes/adducts were considered. Metabolite set enrichment analysis (MSEA) using over representation analysis (ORA) was used to highlight key biochemical pathways.

## Results

**Figure 1:**
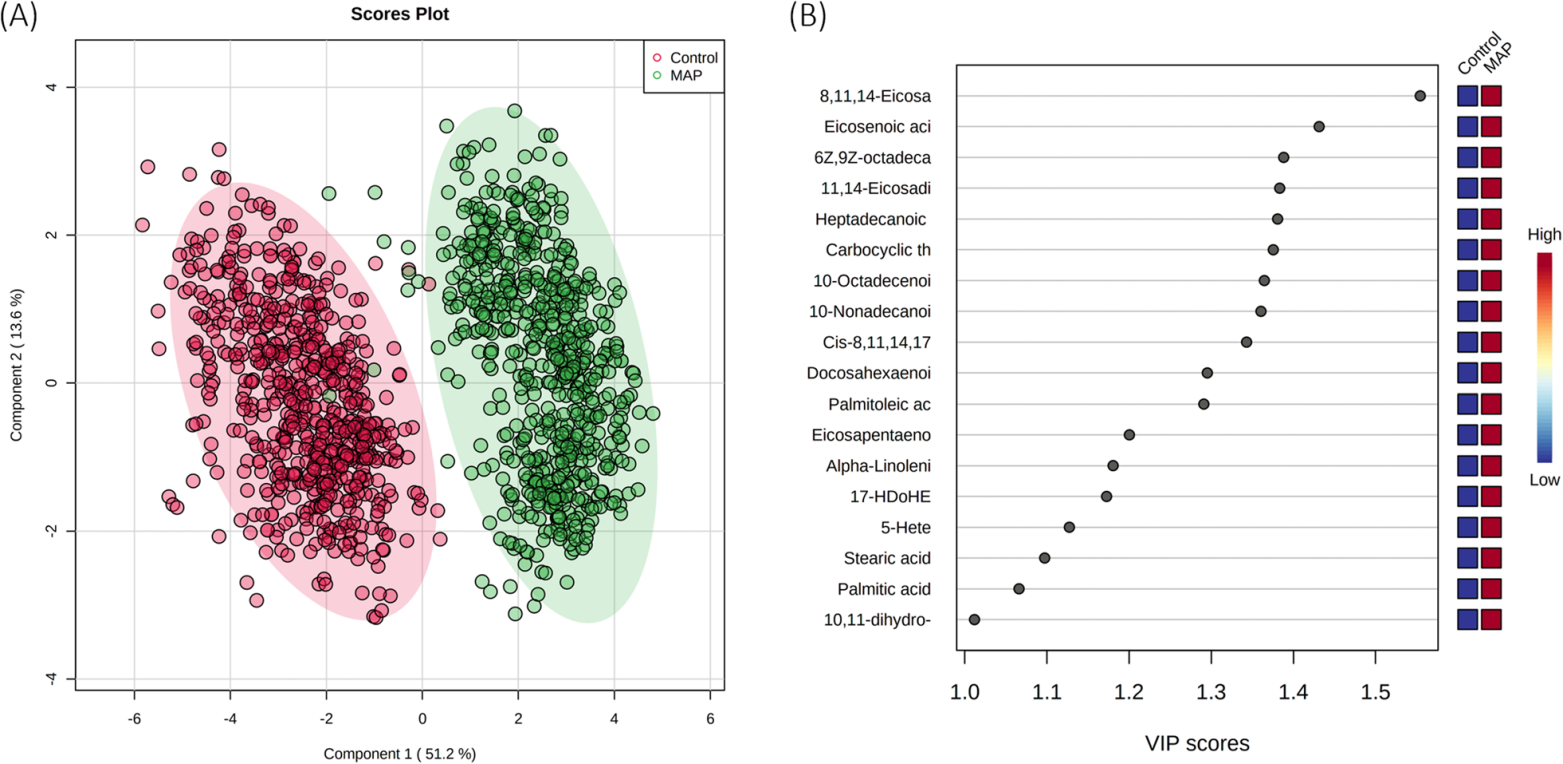
**(A) PLS-DA and (B) VIP score plots.** Plots were produced by PLS-DA of metabolites differentially expressed in MAP-inoculated and control heifer calves between 1-month and 19-months of age in the combined ionization mode m/z. The light red and green ellipses represent 95% confidence intervals.

Serum samples were profiled by FIE-HRMS and the derived spectra assessed by PLS-DA. PLS-DA of the data from both positive and negative ionisations indicted a clear differentiation between MAP-inoculated and control heifer calves (Figure 1A). The sources of variation most utilised in the PLS-DA were indicated using a VIP plot (> 1.0) based on two components (Figure 1B). This indicated that the eicosanoid, 8.11,14 eicosatrienoic acid (C20:4) was the most notable discriminator between MAP-inoculated and control heifer calves. Indeed, other eicosanoids were also shown to have fairly high VIP scores, for example, eicosenoic acid (20:1) and 6Z, 9Z-octadecadienoic acid (C18:2).

Subsequently, the derived metabolomes were assessed using time series analysis, adjusted for FDR, two-way ANOVA. Significant metabolites were identified, and all were significantly affected by time and MAP inoculation status*time (*p* < 0.05) (Additional file 1). Out of 34 identified metabolites, 25 were fatty acyls and notable subclass included; fatty acids and conjugates (16), eicosanoids (6) and linoleic acid and derivatives (3) (Additional file 2).

**Figure 2:**
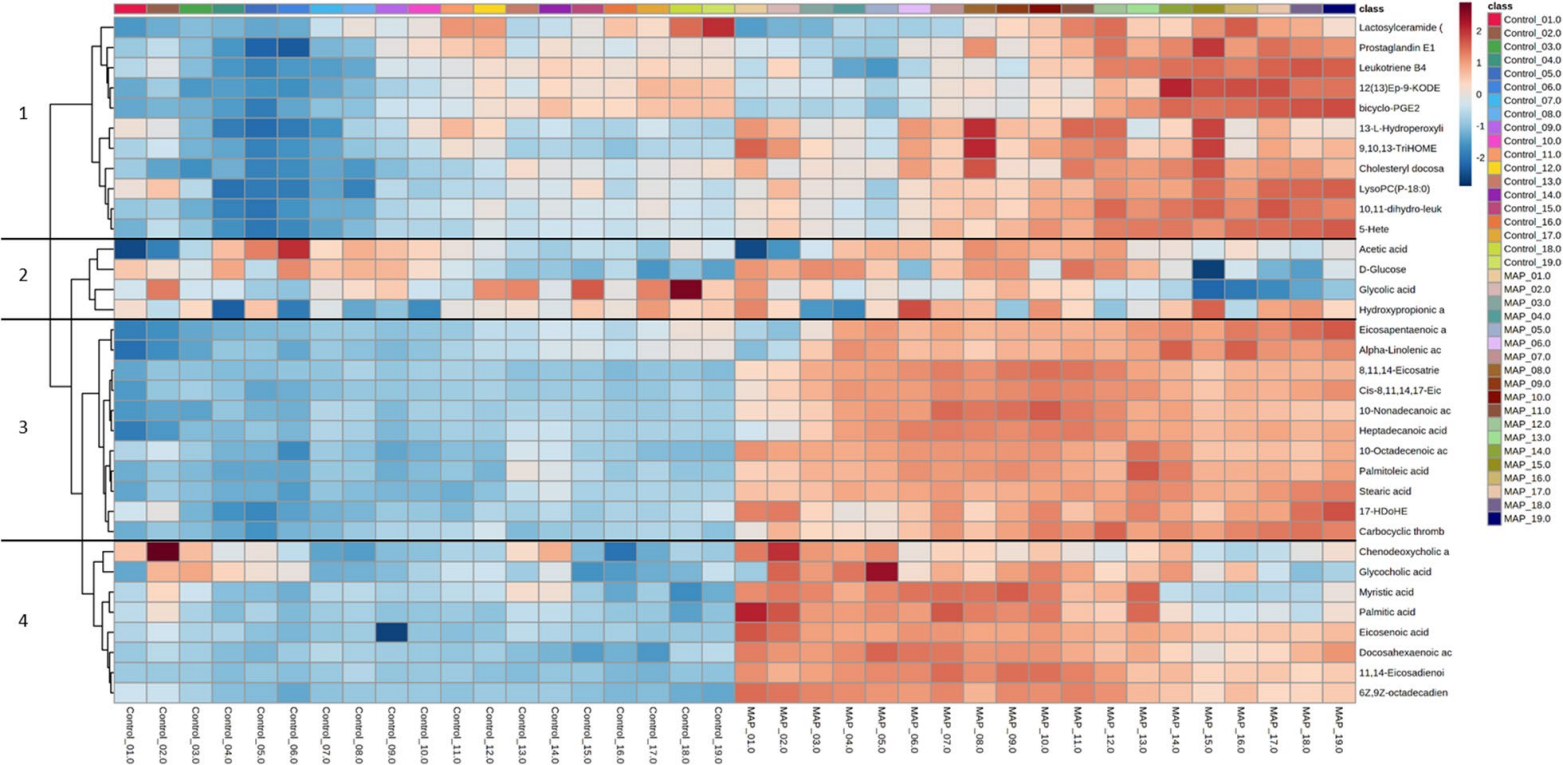
**HCA of significant metabolite changes in serum differentiating between MAP-inoculated and control heifer calves.** Heifer calves were aged between 1-month and 19-months of age. Major patterns of metabolite accumulation during the experiment are indicated by the numbered clades (1,2,3 and 4).

Significantly altered metabolite changes were visualised using a heat map (Figure 2). The accumulation of identified metabolites was significantly higher in MAP-inoculated heifer calves compared to controls. However, the timings of metabolite accumulations differed between clades, whereby clades are defined as groups of metabolites which demonstrate comparable levels over time. Some changes were seen quickly following MAP inoculation (within 1 month) and remained elevated throughout the timeframe of the experiment. These metabolites are included in Figure 2 clade 3 and included alpha linolenic acid, 8.11,14 eicosatrienoic acid and stearic acid. The elevation of other metabolites (Figure 2, clade 4) was equally rapid following MAP inoculation but started to decrease after 12 months. Examples of metabolites in these categories included the fatty acids; myristic and palmitic acid, as well as 6Z, 9Z-octadecadienoic acid (C18:2). Other metabolites were only elevated after 6 to 12 months and then remained higher (Figure 2, clade 1) and include important eicosanoids, such as prostaglandin E1 and leukotriene B4. The eicosanoid, thromboxane A2, was more rapidly elevated and was found in Figure 2, clade 3. Interestingly, many eicosanoid levels in the sera of control heifer calves shown in Figure 2 clade 1, exhibited some slight increases over time, possibly due to heifer growth and development.

**Figure 3:**
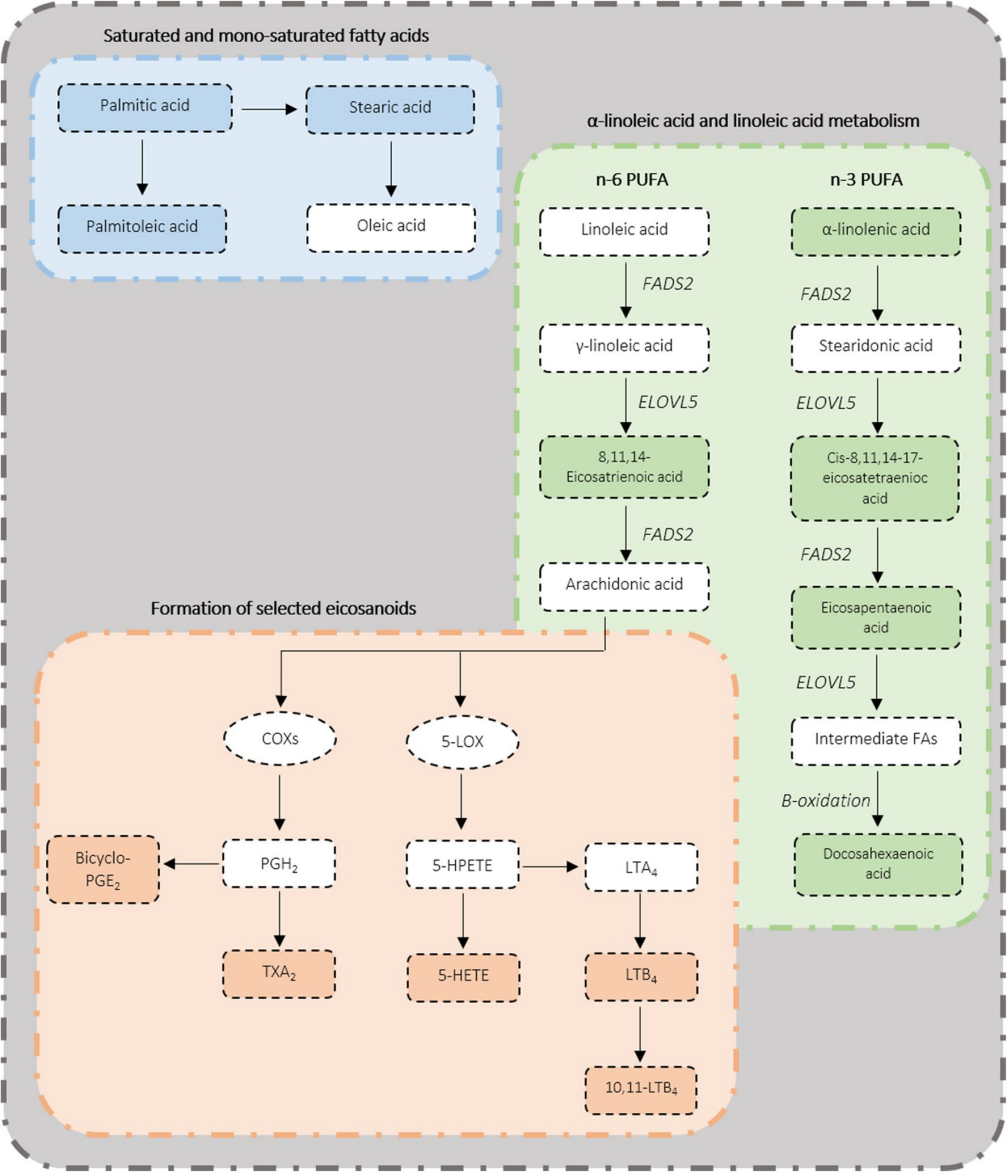
**The metabolomic impact of MAP on selected metabolites.** Metabolites include; selected unsaturated fatty acids, metabolites located within alpha-linolenic acid and linoleic acid metabolism and selected eicosanoids. Coloured boxes = identified metabolites, white boxes = metabolites which were not identified. FADS1 = fatty acid desaturase 1, FADS2 = fatty acid desaturase 2, ELOV5 = elongation of very long chain fatty acids protein 5, COX = cyclooxygenase, 5-LOX = 5-lipoxygenase, PGH2 = prostaglandin H2, 5-HPETE = 5-hydroperoxyeicosatetraenoic acid, 5-HETE = 5-hydroxyeicosatetraenoic acid, TXA2 = carbocyclic thromboxane A2 LTA4 = leukotriene A4, LTB4 = leukotriene B4, 10, 11-LTB4 = 10,11-dihydro-leukotriene B4.

**Figure 4:**
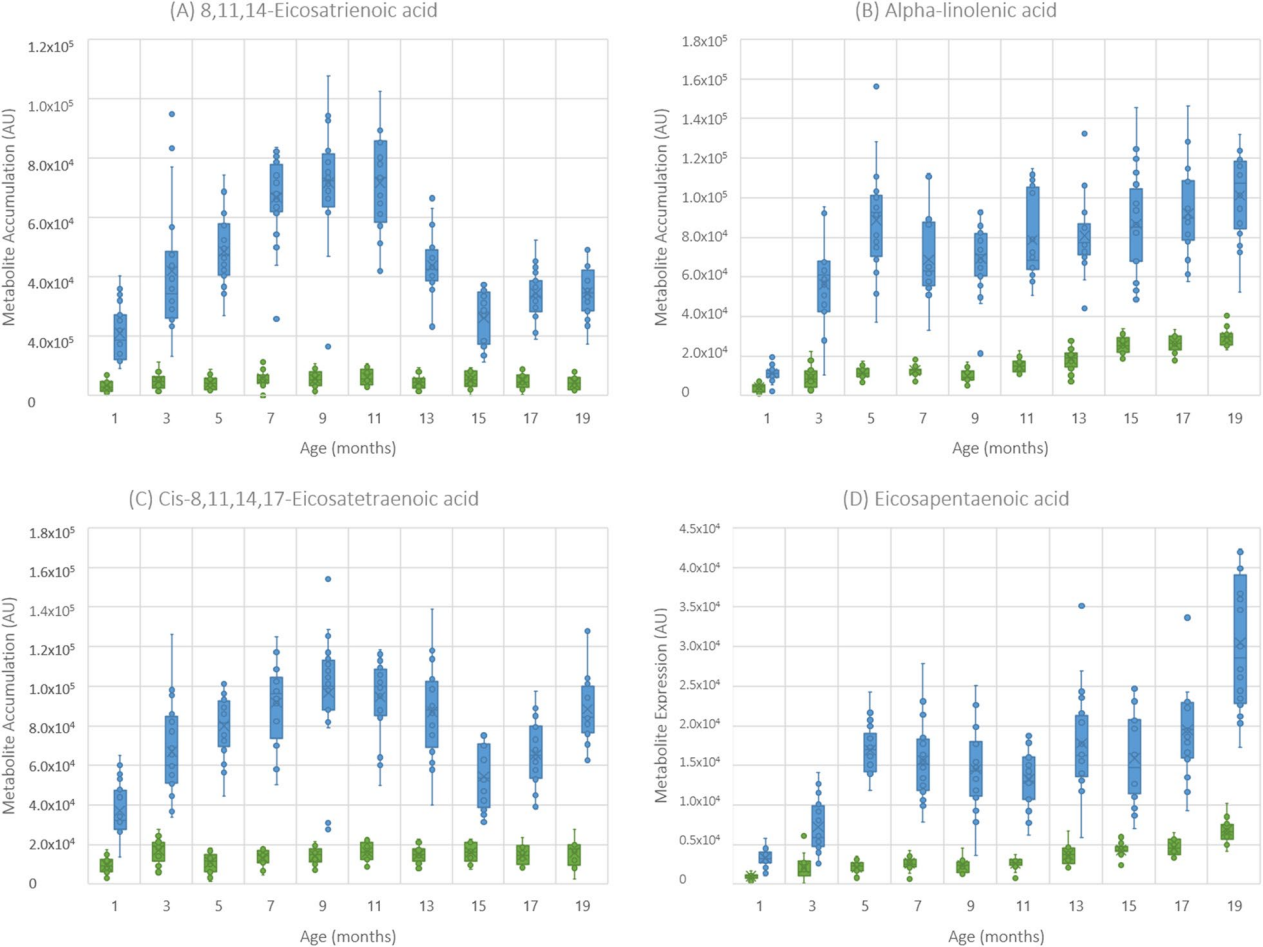
**Box and whisker plots of metabolites which display minimal overlapping between group.** **A** 8,11,14-eicosatrienoic acid; **B** alpha-linoleic acid; **C** cis-8,11,14,17-eicosatetraenoic acid; **D** eicosapentaenoic acid. Blue boxplots = MAP-inoculated heifer calves, green boxplots = control heifer calves.

To better visualise the biological events indicated by these metabolomic changes, identified metabolites were related to different pathways by MSEA using ORA analysis. This indicated that alpha-linoleic acid and linoleic acid metabolism was the only pathway to be significantly (*p* < 0.05) affected by MAP inoculation status (Additional file 3). This pathway is depicted schematically in Figure 3, where the metabolites identified in our study are indicated. To further illustrate changes in the pathway, the accumulation patterns of individual metabolites (8, 11, 14-eicosatrienoic acid, alpha-linoleic acid, *cis*-8, 11, 14, 17-eicosatetraenoic acid and eicosapentaenoic acid) are plotted (Figure 4). Thus, elevated levels of alpha-linoleic acid derivatives was linked to increased levels of docosahexaenoic acid. This metabolite is shown in Figure 2, clade 4, indicating that its levels decreased > 11 months following MAP infection. However, the linoleic acid derivative pathway, linked to cyclooxygenase (COX) and lipoxygenase (LOX)-dependent pathway, was active as shown by the detection of many prostaglandin, thromboxane and leukotriene derivatives. These metabolites increased more slowly following MAP inoculation (Figure 2, clade 1).

Excluding docosahexanoic acid, whereby the inter-quartile ranges of experimental groups overlapped 15 months post MAP inoculation (Additional file 4), metabolites identified within the alpha-linoleic acid and linolenic acid metabolism showed no evidence of increases in control heifer calves over time (Figure 4), suggesting that these changes were intimately associated with MAP inoculation. It was also noted that, the linoleic pathway metabolites, 10-octadecenoic acid and heptadecanoic acid showed no increases in control heifer calves over time (Additional file 5). Consequently, RF and AUC assessments were used to determine the potential of these six metabolites as biomarkers for MAP infections. RF assessments across all ages indicated classification errors for MAP-inoculated and control heifer calves of only 0.00781 and 0.00313, respectively (Table 1). Moreover, AUC assessments of these metabolites at 19-months of age suggested individual metabolite AUC values of 1.0 (C.I 0.913–0.999) (Additional file 2) and a combined AUC value of 0.999 (C.I 0.992–1.000), as well as sensitivity and specificity values of 99.2% and 99.6%, irrespective of age.

**Table 1:** **Cross validation results of the random forest assessments.** These assessments classified serum samples from heifers between 1-month and 19-months of age as MAP-inoculated or control based on metabolite levels of 8,11,14-eicosatrienoic acid, 10-octadecenoic acid, alpha-linolenic acid, cis-8,11,14,17-eicosatetraenoic acid, eicosapentaenoic acid and heptadecanoic acid

	MAP-inoculated	Control	Class error
MAP-inoculated	638	2	0.00313
Control	5	635	0.00781

**Figure 5:**
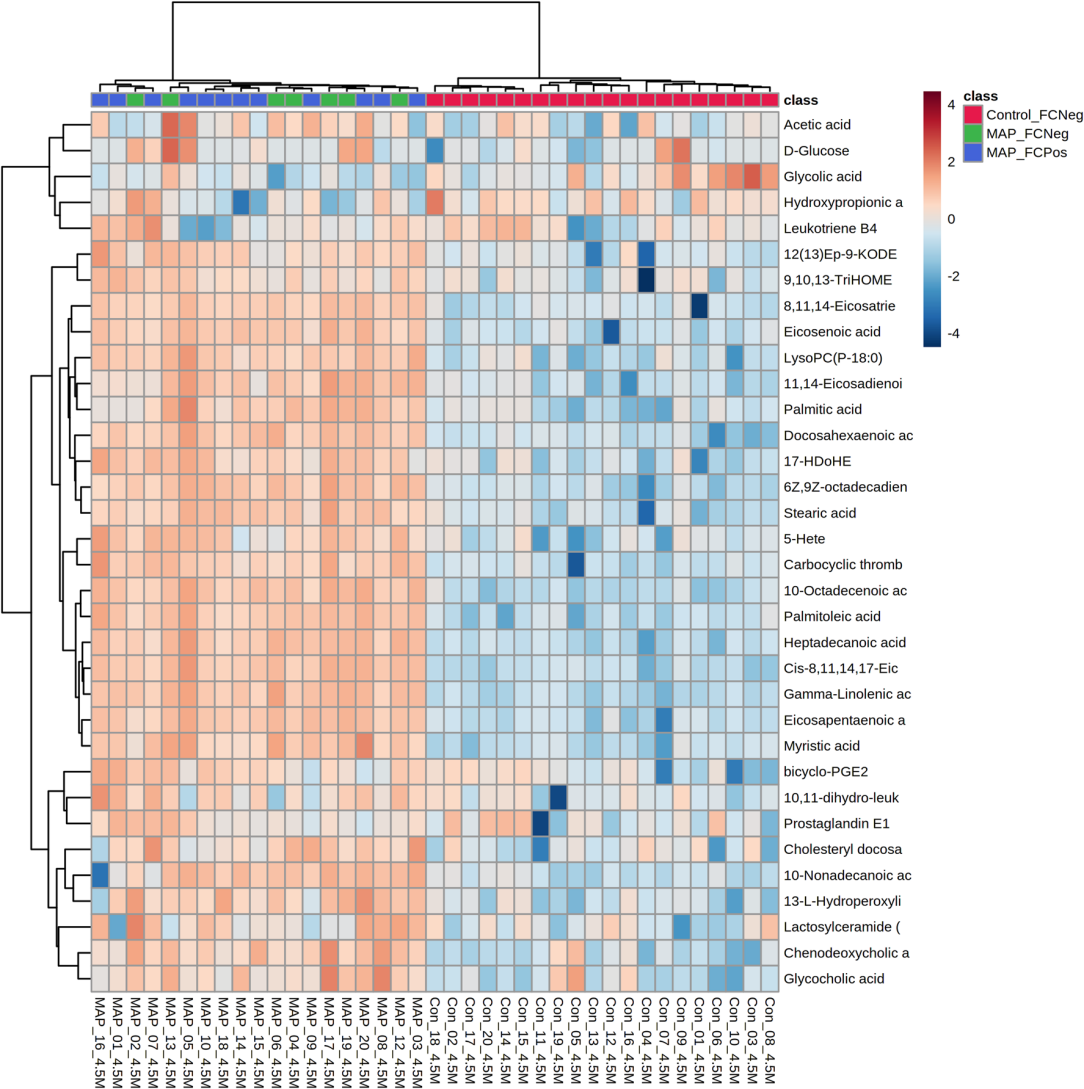
**HCA of the metabolites changes alongside faecal culture results at 4.5-months of age.** The metabolite changes are those which differentiate between MAP-inoculated and control heifer calves in the combined ionisation mode *m/z*.

**Figure 6:**
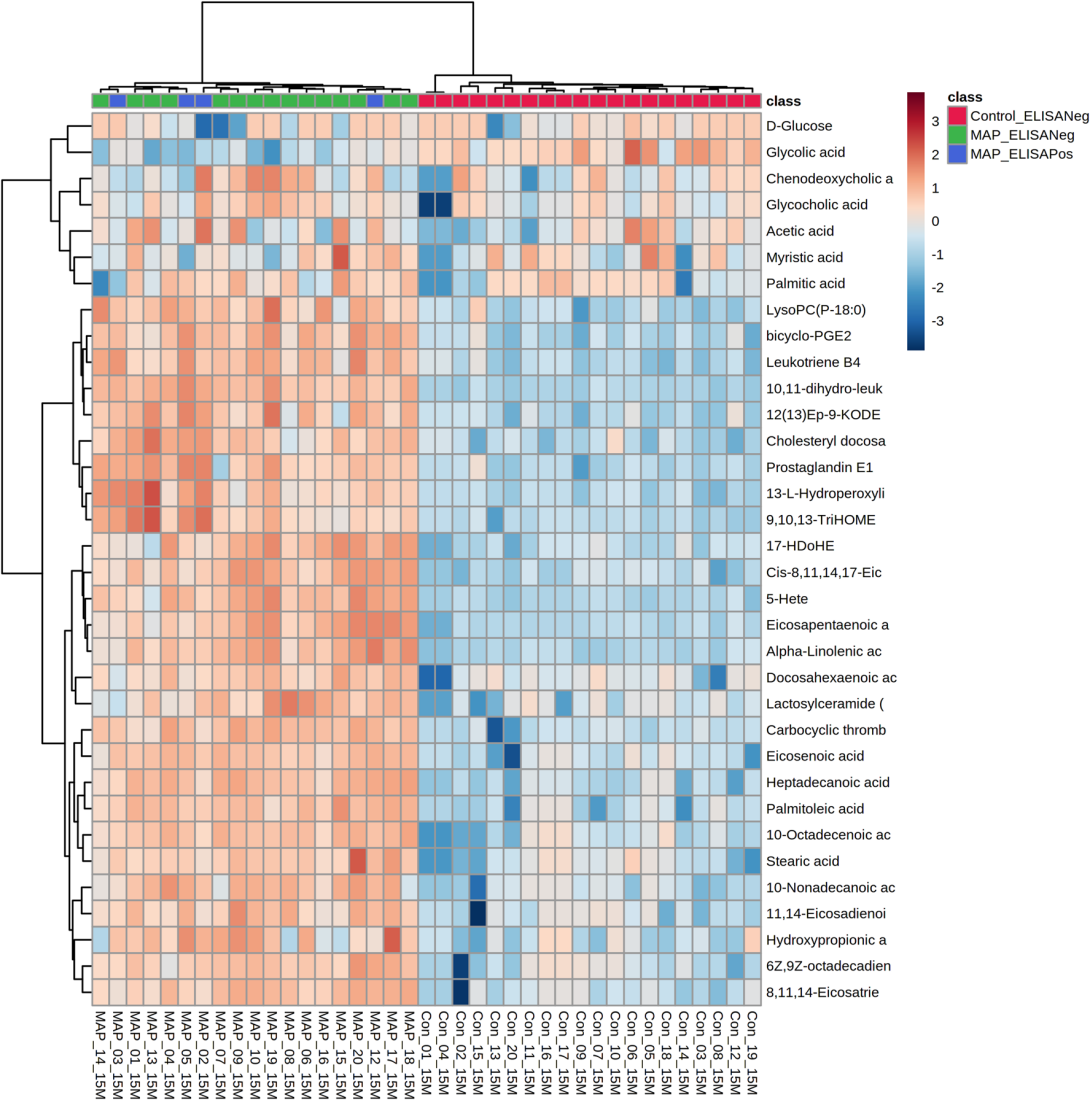
**HCA of the metabolite changes alongside serum ELISA results at 15-months of age**. The metabolite changes are those which differentiate between MAP-inoculated and control heifer calves in the combined ionisation mode *m/z*.

Additionally, we compared the ability of identified metabolites, faecal culture and serum ELISA tests to differentiate between experimental groups. Although our metabolites were unable to differentiate between heifer calves based on faecal culture or serum ELISA status (Figures 5 and 6), our metabolites better discriminated between experimental groups based on MAP inoculation status.

## Discussion

Johne’s disease, caused by MAP, has a significant impact on cattle health alongside severe economic consequences. Current diagnostic tests for MAP, including faecal PCR, serum ELISA [8] and IFN-γ tests [9] rely on detecting a cell mediated immune (CMI) response, antibody production or bacterial shedding. As a result, they perform poorly between the initial CMI response and the sub (clinical) stages of infection [22]. The application of omic approaches have suggested that they could be a potential source of new biomarkers with sensitivity and specificity values reported ranging between 82 and 94% [11, 12]. However, no omic study has assessed MAP infection associated changes in youngstock. Our previous metabolomic study identified 33 metabolites which were differentially accumulated within naturally MAP-infected heifer calves, including 5 metabolites which were elevated throughout the study [16]. In this study, we examined experimentally MAP-inoculated heifer calves to more clearly assess early changes occurring following infection and to validate the previously observed metabolites changes in an analogous study.

Our findings identified 11 metabolites in MAP-inoculated heifer calves that were also observed in our Taylor et al. [16] study which focused on natural MAP infections. The prominence of metabolites from the fatty acyl class and fatty acids and conjugates subclass, alongside the eicosanoid subclass was notable in both studies (Additional file 2) [16]. However, there was an absence of metabolites belonging to the amino acids, peptides and analogues subclass in our current study (Additional file 2). Changes to bioenergetic and uraemic pathways also seem specific to naturally infected heifer calves [16]. These changes may be attributable to differences in bacterial load, as indicated by the faecal culture results. Taylor et al. [16] noted MAP-positive faecal culture results within all MAP-positive heifers. In contrast, only 13 out of 20 MAP-inoculated heifer calves were MAP faecal culture positive within our current study. Thus, implying that a higher bacterial load is required to observe bioenergetic and uraemic changes, compared to fatty acyl related changes. In contrast, fatty acyls changes appear to be consistent in both naturally and experimentally MAP inoculated heifer calves studies. This could support their importance as potential immunomodulators.

Moreover, although Taylor et al. [16] reported that accumulations of leukotriene B4, bicyclo prostaglandin E2, itaconic acid, 2-hydroxyglutaric acid and N6-acetyl-L-lysine were significantly elevated throughout the study, accumulations of the majority of metabolites fluctuated within MAP infected and control heifers up to 13 months of age. In our current study, eicosanoids also followed this pattern (clade 1, Figure 2). However, fatty acids and conjugates demonstrated minimal fluctuations (clade 3, Figure 2). These observations may reflect MAP inoculated heifer calves receiving nine doses of 20 g of faeces, as opposed to being naturally infected. Fecteau et al. [23] and Kaevska et al. [24] observed significant differences within the faecal microbiota of MAP shedding cattle, compared to non-shedding cattle. Likewise, Derakhshani et al. [25] noted the influence of rumen microbiota on functional pathways, such as; carbohydrate, lipid and amino acid metabolism. More recently, Umanets et al. [26] demonstrated how intestinal microbiota can be utilised to predict the severity of future MAP shedding with AUC accuracies between 0.91 and 0.92 for cattle aged between 12 and 24 months of age. Thus, repeat inoculations of MAP culture positive faeces may aid the establishment of a MAP permissive microbiome.

Our previous examination of MAP effects on the sera in naturally infected heifer calves, had indicated the prominence of fatty acid changes [16] and this was again the case in this study. Accumulations of palmitic acid (C16:0) and stearic acid (C18:0) were observed in naturally and experimentally MAP-inoculated heifer calves (Figure 2). Such an observation was partially in agreement with those of Tata et al. [15] who also targeted palmitic acid as a MAP-infection associated change although not stearic acid. In another MAP focused metabolomic study, neither of these metabolites were identified [14]. Although, this could be seen to reduce the validity of our observations, these differences may be attributable to a mixture of biological factors, such as; MAP dose and bacterial strain, and our use of a direct infusion MS approach, as opposed to NMR spectrometry. Equally, there is independent corroboration of our observations in a transcriptional assessment of MAP-infected sheep. Thus, the expression of FABP3 and PDK4, which are associated with the oxidation of palmitic acid, as well as, FABP3 and ORL1, which are linked to the uptake of palmitic acid, were elevated in paucibacillary and resilient MAP-infected sheep, respectively [27]. It was not possible to assess how far these changes reflected metabolic events occurring in either the heifer calves or in the MAP organisms or both combined. It should be noted that palmitic acid and stearic acid are common biochemical features o*f Mycobacterium* [28, 29]. To define the relative origins of particular metabolites will require the use of such as isotopically labelled MAP in further infection experiments as demonstrated in other pathosystems [30].

The likely increased depth of the direct infusion MS approach could have led our targeting of the alpha-linolenic acid (C18:3) and linoleic acid (C18:2) metabolism. This pathway was not identified in other MAP-focused metabolomic studies [14, 15] but was significantly affected by MAP inoculation status in our current (Additional file 3) and previous study [16]. The metabolites that formed part of the alpha-linolenic acid/linoleic acid (C18:2) pathway were previously suggested to be potential biomarkers for MAP infection in heifer calves [16]. This underlined the validity of changes in 8, 11, 14-eicosatrienoic acid, cis-8, 11, 14–17-eicosatetraenioc acid, eicosapentaenoic acid and docosahexaenoic acid as early biomarkers for MAP infection. Two patterns of alpha-linolenic acid/linoleic acid metabolite accumulation was observed. Most metabolites in this pathway increased over time (Figure 2), however, docosahexaenoic acid showed a rapid increase, followed by a decline in levels after ~10 months. This raises the question as to the clinical significance of these differential accumulation patterns.

Metabolites derived from alpha-linolenic acid are n-3 polyunsaturated fatty acids (PUFAs) (where the double bond is three atoms away from the terminal methyl group) and metabolites derived from linoleic acid are n-6 PUFAs (double bond is six atoms away from the methyl group). Linoleic acid feeds into the production of arachidonic acid (C20:4) from which a range of pro-inflammatory eiconsoids are derived, for example, prostaglandins and leukotrienes. n-6 PUFA, such as eicosapentaenoic and docosahexanoic acids (as detected in MAP infections) will inhibit arachidonic metabolism. However, it is very much an over-simplification to suggest that n-3 PUFAs minimise inflammation and n-6 PUFAs promote inflammation. For example, prostaglandin E2 has both a classic pro-inflammatory and an anti-inflammatory role [31]. This stated, the decreases in docosahexanoic acid (Figure 2, clade 4) at 10 to 12 months post MAP inoculation is followed by increases in leukotriene B4 and prostaglandins. Thus, could indicate that an initial phase in responses to MAP where anti-inflammatory responses are dominant, but then at later phases, inflammation dominates. The potential roles of n-3/n-6 PUFAs ratio in MAP infections has not been assessed but there may be relevant parallels from *M. tuberculosis* infections. *M. tuberculosis* colonisation was increased in the spleens of guinea pigs fed an n-3 PUFA based diet, compared to n-6 PUFA based or control diets [32, 33]. Similarly, *M. tuberculosis* infected guinea pigs fed an n-3 PUFA based diet exhibited increased *M. tuberculosis* pulmonary colonisation compared to controls [34]. Such studies suggest that the observed increased n-3 PUFAs (cis-8, 11,14,17-eicosatetraenoic acid, eicosapentaenoic acid and docosahexaenoic acid) encourage MAP colonisation, possibly linked to an anti-inflammatory role.

Arachidonic acid discourages *M. tuberculosis* growth by stimulating the secretion of TNF-α but eicosapentaenoic acid supported colonisation by inhibiting TNF-α secretion [35]. Anes et al. [36] observed similar effects with *M. avium* whereby n-3 PUFAs stimulated growth. In further support of this, incubating macrophage-like J77A4.1 cells with conjugated docosahexaenoic acid suppressed the production of pro-inflammatory cytokines (TNF-α, IL-6 and MCP-1) and co-stimulatory molecules (CD40 and CD86) [37]. Consequently, our findings indicate that levels of early n-3 PUFAs and mycobacterial colonisation increase simultaneously. However, our hypothesis may be too simplistic as *M. tuberculosis* infected mice on an n-3 PUFA enriched diet demonstrated elevated pathogen killing [35] and those with a deficient diet showed reduced levels of pro-inflammatory cytokines (IL-6, IL-1α, MCP1, MIP1 and RANTES) and elevated levels of anti-inflammatory cytokines (IL-4 and growth factor GMCSF) [38]. Clearly, further MAP-focused studies are required to define the role of the early n-3 PUFA increases. Previous studies suggested that lipid-related metabolomic [14] and lipidomic [39] changes were indicative of lipid mobilisation that occurred in response to a reduced absorptive capability of the small intestine. However, this is an unlikely cause of our observed lipid-related changes as the heifer calves were asymptomatic throughout the study.

n-6 PUFA increases via the arachidonic acid-derived eicosanoid pathway were one of the most prominent subclasses of metabolites changing on MAP infection (Additional file 2). Many important eicosanoids increased 11 months post MAP inoculation although carbocyclic thromboxane A2 appear to be increasing at earlier time point (Figure 2). Crucially, two of these metabolites, leukotriene B4 and bicylo-PGE2 were identified in our previous study underlying their validity as biomarkers [16]. Eicosanoids processing from arachidonic acid can follow one of two enzymatic routes; the cyclooxygenase (COX) mediated route produces prostaglandins and thromboxane’s, and the lipoxygenase (LOX) mediated route produces hydroxyeicosatetraenoic acids (HETEs) and leukotrienes [40]. Our analyses showed that the metabolite end-points of both routes accumulated at later points of MAP infections (Figure 3). As these are mostly involved in mediating inflammation [41], this was consistent with inflammation dominating MAP infection after 11 months, probably after the initial phase where MAP was suppressed. Steps which could be suppressed could include neutrophil migration and recruitment of CD4 and CD8 T lymphocytes which have been linked to LOX-derived metabolites promotion [42, 43]. There is some evidence for this from transcriptomic assessments where the LOX pathway, as indicated by ALOX15, is downregulated in both MAP positive cows [44] and calves 6 months post MAP inoculation [45]. Further, ALOX5 was down-regulated in primary monocyte-derived macrophages in MAP positive cows [46]. This would align with the delayed accumulation of LOX-dependent metabolites, including 5-HETE, 10, 11-dihydro-leukotriene B4 and leukotriene B4 in our metabolomic study (Figure 2).

Considering the COX pathway, it seems likely that this does not wholly conform to the pro-inflammatory phase model in our MAP infection experiment. The literature suggests that expression of the COX2 encoding gene, PTGS2, is rapidly increased in primary monocyte-derived macrophages from MAP positive cows at 8 to 24 h post-infection [46]. Outside of MAP studies, *M. bovis* elicits elevated levels of COX-dependent prostaglandin E_2_ [47], as well as increased expression of prostaglandin E synthase [48]. We did observe an increase in thromboxane A2 in first few months following MAP infection. Thromboxane A2 is produced by platelets and vaso-constrictive and prothrombotic properties, so is not directly inflammatory. Further, thromboxane A2 is unstable so that its production must be persistent to allow its detection in our assays [49]. It may be that early COX metabolism feeds into this sub-pathway, rather than more proinflammatory COX products such as prostaglandin E_1_ and bicyclo-PGE2 which were observed at later timepoints of MAP infection. However, we also observed later increases in prostaglandin E_2_ which increases levels of the regulatory cytokine IL-10 although decreasing T-cell proliferation and Th1 cytokine production but and immunosuppressive programmed death ligand 1 [50]. The absence of clear mechanistic trends to emerge from our eicosanoid assessments could be as a result of our focusing on sera which represented the pooling of metabolites arising from a range of cell and tissue specific effects. Further research assessing immune system parameters, including in specific cell types, are required to define the influence of LOX-dependent and COX-dependent metabolites on inflammatory events and the immune system.

Metabolomic analysis of experimentally MAP-inoculated and control heifer calves using untargeted FIE-HRMS successfully differentiated between experimental groups between 1- and 19- months post MAP inoculation. Broadly, two main types of response were observed, a rapid increase (within 1 month) of saturated fatty acids and some n-3 PUFA that could promote colonisation and suppress inflammation, and a later accumulation of fatty acids including n-6 PUFA and COX products that could indicate a more pro-inflammatory, immune-modulatory phase. Crucially, six fatty acyls were able to differentiate between experimental groups throughout the study. Moreover, several metabolites that were suggested to be biomarkers for MAP in naturally infected heifer calves were also observed in this study where heifer calves were experimentally inoculated. Future work could include examining immune system parameters alongside these targeted metabolites.

## Supplementary Information

**Additional file 1. Metabolites that significantly change following MAP inoculation**. *P *values are indicated. Details of the identified metabolites along with *p*-values of MAP status, time and MAP status*time.

**Additional file 2. The sub (classes) and AUC values of metabolites differentially expressed in MAP-inoculated heifer calves at 19-months of age. **Details of the identified metabolites including; metabolite ionisation modes, class, subclass, AUC, *P*-value and Log_2_ (FC) at 19-months of age.

**Additional file 3. Significantly enriched pathways in MAP-inoculated heifer calves in the combined ionisation mode. **The output of MSEA using ORA demonstrating the significantly enriched pathways in MAP-inoculated heifer calves in the combined ionisation mode.

**Additional file 4. Box and whisker plots of metabolites which display minimal overlapping between groups, MAP-inoculated and control heifer calves, between 1-month and 19-months of age.** (A) 10-Octadecenoic acid; (B) heptadecanoic acid. Blue boxplots = MAP-inoculated heifer calves, green boxplots = control heifer calves.

**Additional file 5. Box and whisker plots of docosahexaenoic acid which displays minimal overlapping between groups, MAP-inoculated and control heifer calves, between 1-month and 19-months of age. **Blue boxplots = MAP-inoculated heifer calves, green boxplots = control heifer calves.

## Data Availability

The datasets generated and/or analysed during the current study are available in the Mendeley Data repository [51].
