# Supplementary material for: Metabolomic changes in polyunsaturated fatty acids and eicosanoids as diagnostic biomarkers in Mycobacterium avium ssp. paratuberculosis (MAP)-inoculated Holstein–Friesian heifers
Source: Vet Res. 2022 Sep 2;53:68. doi: 10.1186/s13567-022-01087-0 (PMC9440510; doi:10.1186/s13567-022-01087-0)
Supplement: Supplementary file 1 — Additional file 1. Metabolites that significantly change following MAP inoculation. P values are indicated. Details of the identified metabolites along with p-values of MAP status, time and MAP status*time. [file 13567_2022_1087_MOESM1_ESM.docx]

| **Additional file 1** **Metabolites that significantly changes following MAP inoculation.** *P*-values are indicated. | | | |
| --- | --- | --- | --- |
| **Common Metabolite Name** | **MAP Status** | **Time** | **Interaction** |
| 10,11-dihydro-leukotriene B4 | 4.76 × 10^-16^ | 3.49 × 10^-200^ | 1.84 × 10^-19^ |
| 10-Nonadecanoic acid* | 4.93 × 10^-31^ | 7.66 × 10^-28^ | 1.47 × 10^-9^ |
| 10-Octadecenoic acid | 5.14 × 10^-35^ | 3.17 × 10^-35^ | 7.40 × 10^-20^ |
| 11,14-Eicosadienoic acid | 2.38 × 10^-34^ | 2.03 × 10^-16^ | 9.47 × 10^-9^ |
| 12(13)Ep-9-KODE | 7.24 × 10^-10^ | 1.75 × 10^-264^ | 5.52 × 10^-37^ |
| 13-L-Hydroperoxylinoleic acid | 4.60 × 10^-12^ | 1.02 × 10^-108^ | 2.81 × 10^-48^ |
| 17-HDoHE* | 3.22 × 10^-23^ | 5.94 × 10^-137^ | 1.16 × 10^-7^ |
| 5-Hete | 2.09 × 10^-21^ | 5.85 × 10^-280^ | 6.39 × 10^-19^ |
| 6Z,9Z-octadecadienoic acid | 1.82 × 10^-36^ | 9.77 × 10^-43^ | 7.45 × 10^-10^ |
| 8,11,14-Eicosatrienoic acid* | 2.52 × 10^-36^ | 1.51 × 10^-54^ | 1.00 × 10^-11^ |
| 9,10,13-TriHOME | 5.08 × 10^-15^ | 1.03 × 10^-73^ | 3.39 × 10^-26^ |
| Acetic acid | 3.58 × 10^-4^ | 0.00 × 10^0^ | 9.85 × 10^-26^ |
| Alpha-Linolenic acid | 8.53 × 10^-32^ | 0.00 × 10^0^ | 1.29 × 10^-51^ |
| Bicyclo-PGE2* | 5.14 × 10^-10^ | 0.00 × 10^0^ | 2.38 × 10^-8^ |
| Carbocyclic thromboxane A2 | 3.96 × 10^-27^ | 2.41 × 10^-129^ | 9.66 × 10^-9^ |
| Chenodeoxycholic acid | 8.47 × 10^-5^ | 2.87 × 10^-42^ | 6.51 × 10^-9^ |
| Cholesteryl docosahexaenoic acid | 4.17 × 10^-25^ | 3.63 × 10^-52^ | 5.94 × 10^-27^ |
| Cis-8,11,14,17-Eicosatetraenoic acid* | 1.64 × 10^-31^ | 4.27 × 10^-48^ | 1.06 × 10^-19^ |
| D-Glucose | 3.21 × 10^-4^ | 2.99 × 10^-34^ | 1.32 × 10^-10^ |
| Docosahexaenoic acid* | 4.19 × 10^-25^ | 1.65 × 10^-46^ | 3.74 × 10^-9^ |
| Eicosapentaenoic acid* | 5.49 × 10^-27^ | 1.71 × 10^-238^ | 1.87 × 10^-32^ |
| Eicosenoic acid | 1.33 × 10^-29^ | 8.91 × 10^-45^ | 2.71 × 10^-28^ |
| Glycocholic acid | 1.70 × 10^-6^ | 2.78 × 10^-33^ | 3.80 × 10^-17^ |
| Glycolic acid | 9.42 × 10^-5^ | 1.76 × 10^-15^ | 3.84 × 10^-17^ |
| Heptadecanoic acid | 5.98 × 10^-38^ | 9.06 × 10^-154^ | 6.88 × 10^-33^ |
| Hydroxypropionic acid | 3.34 × 10^-8^ | 2.98 × 10^-83^ | 2.46 × 10^-43^ |
| Lactosylceramide (d18:1/16:0) | 3.31 × 10^-3^ | 8.09 × 10^-170^ | 9.38 × 10^-9^ |
| Leukotriene B4* | 2.30 × 10^-8^ | 2.46 × 10^-190^ | 2.30 × 10^-16^ |
| LysoPC(P-18:0) | 1.92 × 10^-15^ | 1.19 × 10^-150^ | 2.04 × 10^-78^ |
| Myristic acid | 2.86 × 10^-23^ | 3.28 × 10^-102^ | 7.52 × 10^-56^ |
| Palmitic acid* | 6.40 × 10^-29^ | 1.22 × 10^-69^ | 2.39 × 10^-22^ |
| Palmitoleic acid* | 1.23 × 10^-33^ | 3.37 × 10^-22^ | 7.90 × 10^-14^ |
| Prostaglandin E1 | 9.58 × 10^-8^ | 9.58 × 10^-143^ | 3.84 × 10^-22^ |
| Stearic acid* | 2.33 × 10^-28^ | 6.29 × 10^-7^ | 8.96 × 10^-3^ |
| * identified by Taylor et al. [16] | | | |
