# Supplementary material for: Metabolomic changes in polyunsaturated fatty acids and eicosanoids as diagnostic biomarkers in Mycobacterium avium ssp. paratuberculosis (MAP)-inoculated Holstein–Friesian heifers
Source: Vet Res. 2022 Sep 2;53:68. doi: 10.1186/s13567-022-01087-0 (PMC9440510; doi:10.1186/s13567-022-01087-0)
Supplement: Supplementary file 2 — Additional file 2. The sub (classes) and AUC values of metabolites differentially expressed in MAP-inoculated heifer calves at 19-months of age. Details of the identified metabolites including; metabolite ionisation modes, class, subclass, AUC, P-value and Log2 (FC) at 19-months of age. [file 13567_2022_1087_MOESM2_ESM.docx]

| Additional file 2 The sub(classes) and AUC values of metabolites differentially expressed in MAP inoculated heifers at 19-months of age. | | | | | | |  |
| --- | --- | --- | --- | --- | --- | --- | --- |
| Class | **Sub Class** | **Common Metabolite Name** | **Mode** | **AUC** | ***p-*value** | **Log_2_(FC)** | |
| Carboxylic acids and derivatives | Carboxylic acids | Acetic acid | Neg | 0.590 | 1.94 × 10^-1^ | -0.138 | |
| Fatty Acyls | Eicosanoids | 10,11-dihydro-leukotriene B4 | Neg | 1.000 | 1.10 × 10^-17^ | -2.435 | |
|  |  | 5-Hete | Neg | 1.000 | 1.47 × 10^-24^ | -2.781 | |
|  |  | Bicyclo-PGE2* | Neg | 0.993 | 7.09 × 10^-12^ | -1.736 | |
|  |  | Carbocyclic thromboxane A2 | Neg | 1.000 | 1.13 × 10^-15^ | -3.379 | |
|  |  | Leukotriene B4* | Neg | 0.983 | 1.82 × 10^-12^ | -1.7132 | |
|  |  | Prostaglandin E1 | Neg | 0.855 | 4.19 × 10^-5^ | -1.067 | |
|  | Fatty acids and conjugates | 10-Nonadecanoic acid* | Neg | 0.965 | 2.44 × 10^-10^ | -2.039 | |
|  |  | 10-Octadecenoic acid | Neg | 1.000 | 1.33 × 10^-17^ | -2.546 | |
|  |  | 11,14-Eicosadienoic acid | Neg | 1.000 | 3.21 × 10^-9^ | -1.915 | |
|  |  | 12(13)Ep-9-KODE | Neg | 0.813 | 9.08 × 10^-5^ | -0.606 | |
|  |  | 17-HDoHE* | Neg | 1.000 | 6.40 × 10^-18^ | -2.295 | |
|  |  | 8,11,14-Eicosatrienoic acid* | Neg | 1.000 | 9.27 × 10^-18^ | -2.990 | |
|  |  | 9,10,13-TriHOME | Neg | 0.973 | 3.07 × 10^-9^ | -1.052 | |
|  |  | Cis-8,11,14,17-Eicosatetraenoic acid* | Neg | 1.000 | 9.34 × 10^-17^ | -2.472 | |
|  |  | Docosahexaenoic acid* | Neg | 1.000 | 5.12 × 10^-17^ | -2.100 | |
|  |  | Eicosapentaenoic acid* | Neg | 1.000 | 1.01 × 10^-20^ | -2.176 | |
|  |  | Eicosenoic acid | Neg | 1.000 | 1.19 × 10^-14^ | -2.522 | |
|  |  | Heptadecanoic acid | Neg | 1.000 | 3.09 × 10^-22^ | -2.398 | |
|  |  | Myristic acid | Neg | 0.930 | 4.74 × 10^-7^ | -0.920 | |
|  |  | Palmitic acid* | Neg | 0.894 | 6.67 × 10^-5^ | -1.228 | |
|  |  | Palmitoleic acid* | Neg | 1.000 | 3.04 × 10^-15^ | -2.322 | |
|  |  | Stearic acid* | Neg | 0.990 | 1.24 × 10^-11^ | -1.842 | |
|  | Linoleic acids and derivatives | 13-L-Hydroperoxylinoleic acid | Neg | 0.830 | 3.25 × 10^-4^ | -0.684 | |
|  |  | 6Z,9Z-octadecadienoic acid | Neg | 1.000 | 2.45 × 10^-9^ | -2.278 | |
|  |  | Alpha-Linolenic acid | Neg | 1.000 | 8.70 × 10^-22^ | -1.771 | |
| Glycerophospholipids | Glycerophosphocholines | LysoPC(P-18:0) | Neg | 1.000 | 9.20 × 10^-13^ | -1.766 | |
| Hydroxy acids and derivatives | Alpha hydroxy acids and derivatives | Glycolic acid | Neg | 0.683 | 2.20 × 10^-2^ | 0.263 | |
|  | Beta hydroxy acids and derivatives | Hydroxypropionic acid | Neg | 0.550 | 5.69 × 10^-1^ | 0.028 | |
| Organooxygen compounds | Carbohydrates and carbohydrate conjugates | D-Glucose | Neg | 0.586 | 8.34 × 10^-2^ | -0.249 | |
| Sphingolipids | Glycosphingolipids | Lactosylceramide (d18:1/16:0) | Pos | 0.965 | 2.82 × 10^-6^ | 1.385 | |
| Steroids and steroid derivatives | Bile acids, alcohols and derivatives | Chenodeoxycholic acid | Neg | 0.698 | 2.18 × 10^-2^ | -0.745 | |
|  |  | Glycocholic acid | Neg | 0.558 | 5.27 × 10^-1^ | -0.108 | |
|  | Steroid esters | Cholesteryl docosahexaenoic acid | Pos | 0.813 | 7.09 × 10^-2^ | -0.926 | |
| 1 = Ionisation mode m/z  2 = Area under the curve  3 = P-value comparing MAP status effect results from t-test using an adjusted *P*-value (FDR) cut-off of 0.05  4 = Log_2_(FC) (fold change) in naturally MAP infected heifers  * = Identified by Taylor et al. [16] | | | | | | |  |
