# Supplementary material for: Metabolomic changes in polyunsaturated fatty acids and eicosanoids as diagnostic biomarkers in Mycobacterium avium ssp. paratuberculosis (MAP)-inoculated Holstein–Friesian heifers
Source: Vet Res. 2022 Sep 2;53:68. doi: 10.1186/s13567-022-01087-0 (PMC9440510; doi:10.1186/s13567-022-01087-0)
Supplement: Supplementary file 3 — Additional file 3. Significantly enriched pathways in MAP-inoculated heifer calves in the combined ionisation mode. The output of MSEA using ORA demonstrating the significantly enriched pathways in MAP-inoculated heifer calves in the combined ionisation mode. [file 13567_2022_1087_MOESM3_ESM.docx]

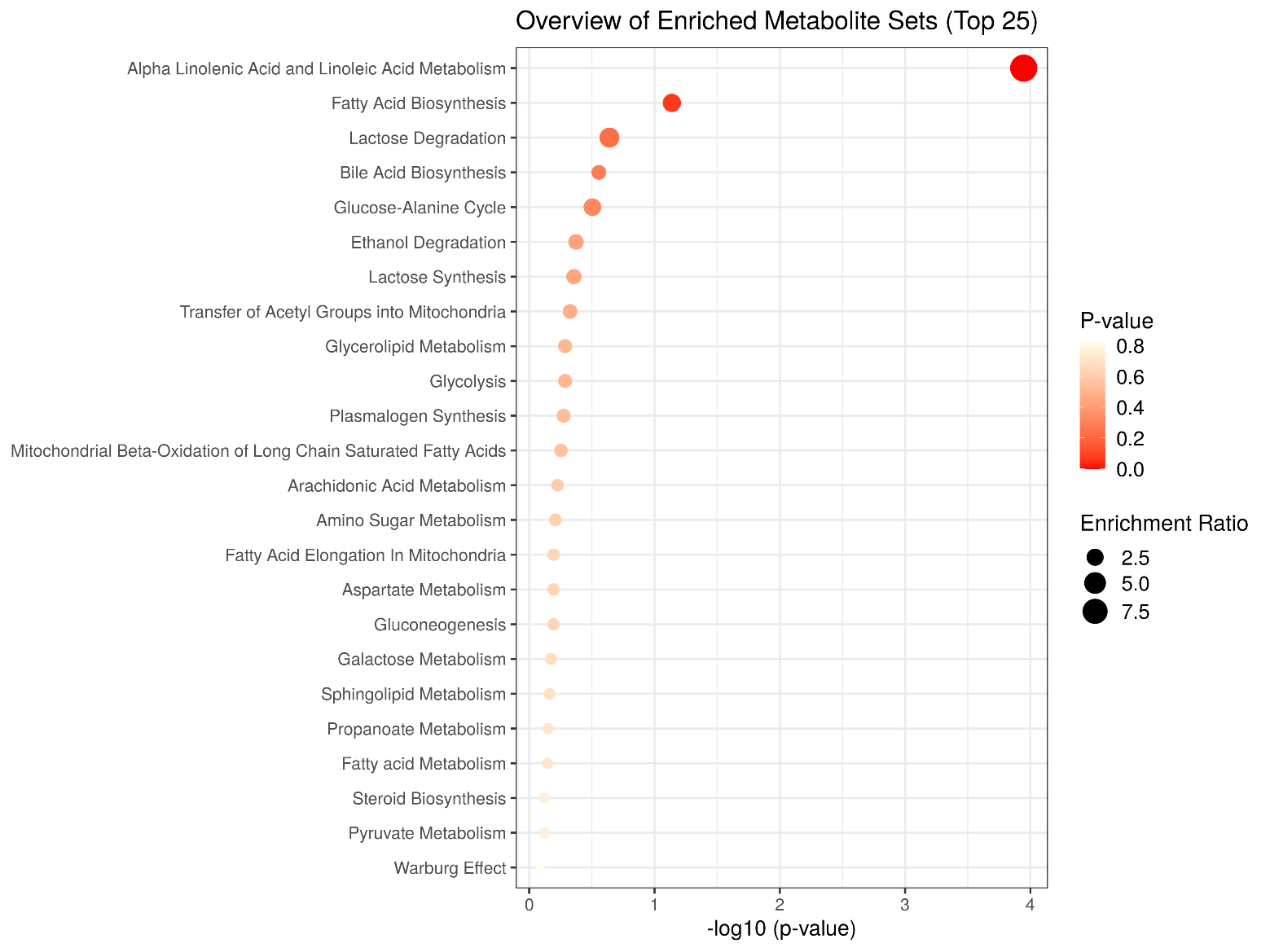


Significantly enriched pathways in MAP-inoculated heifers in the combined ionisation mode.

*p*-value

-log_10_(*p*-value)
