# Supplementary material for: Metabolomic changes in polyunsaturated fatty acids and eicosanoids as diagnostic biomarkers in Mycobacterium avium ssp. paratuberculosis (MAP)-inoculated Holstein–Friesian heifers
Source: Vet Res. 2022 Sep 2;53:68. doi: 10.1186/s13567-022-01087-0 (PMC9440510; doi:10.1186/s13567-022-01087-0)
Supplement: Supplementary file 5 — Additional file 5. Box and whisker plots of docosahexaenoic acid which displays minimal overlapping between groups, MAP-inoculated and control heifer calves, between 1-month and 19-months of age. Blue boxplots = MAP-inoculated heifer calves, green boxplots = control heifer calves. [file 13567_2022_1087_MOESM5_ESM.docx]

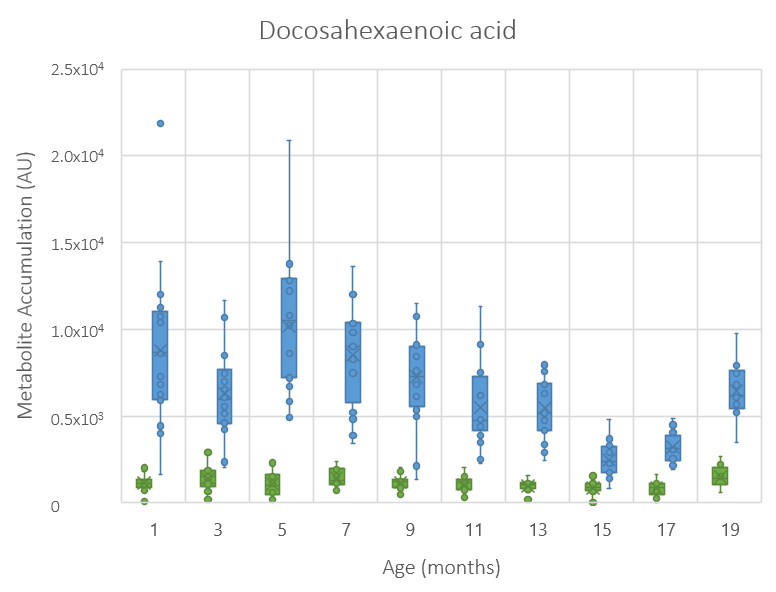


Additional file 5 Box and whisker plots of docosahexaenoic acid which display minimal overlapping between groups, MAP inoculated and control heifers, between 1-month and 19-months of age. Blue boxplots = MAP-inoculated heifers, green boxplots = control heifers.
